# A meta-analysis of students’ readiness assurance test performance with team-based learning

**DOI:** 10.1186/s12909-020-02139-9

**Published:** 2020-07-14

**Authors:** Phan Nguyen Ngoc, Chao-Ling Cheng, Yen-Kuang Lin, Ming-Shun Wu, Jan-Show Chu, Kung-Pei Tang

**Affiliations:** 1 International Master’s Program in Health Care Administration, College of Management, Taipei Medical University, Taipei, Taiwan; 2 Division of Gastroenterology and Hepatology, Department of Internal Medicine, Taipei Municipal Wanfang Hospital, Taipei Medical University, Taipei, Taiwan; 3 Graduate Institute of Data Science, Taipei Medical University, Taipei, Taiwan; 4 Department of Internal Medicine, School of Medicine, College of Medicine, Taipei Medical University, Taipei, Taiwan; 5 Department of Pathology, School of Medicine, College of Medicine, Taipei Medical University, Taipei, Taiwan; 6 Department of Education and Humanities in Medicine, School of Medicine, College of Medicine, Taipei Medical University, 250 Wuxing Street, Xinyi District, Taipei, 11031 Taiwan

**Keywords:** Team-based learning, Readiness assurance test, Meta-analysis, Medical education, Education in healthcare professions

## Abstract

**Background:**

Team-based learning (TBL) is increasingly being utilized across medical fields by engaging students in small group discussions. The readiness assurance test (RAT) is an essential feature that differentiates TBL from problem-based learning (PBL) activity sequences. No publication has discussed differences in the RAT in TBL in medical schools. The purpose of this meta-analysis study was to examine the performance of learners in terms of group RAT (GRAT) and individual RAT (IRAT) scores in TBL for students of healthcare professions.

**Methods:**

Databases, including PubMed and Cochrane were searched using several terms. We assessed the quality of included studies and conducted a meta-analysis.

**Results:**

In total, 11 studies with 1575 participants were identified. Quality assessment scores of these studies ranged 4 ~ 7. Mean GRAT scores were significantly higher than mean IRAT scores (standardized mean difference (SMD) = 2.027, 95% confidence interval (CI) = 1.657 ~ 2.486, *p* heterogeneity < 0.001). Although the test of subgroup differences was insignificant (*p* = 0.113), the nursing-only subgroup showed much better performance in the GRAT than the IRAT (SMD = 2.3CI: 95% CI = 2.0 ~ 2.6, *I*^2^ = 48.77%) compared to the others subgroup which included students from different majors. The subgroup analysis explained the heterogeneity in the overall analysis. Because of inadequate information from these 11 studies, a meta-regression could not explore the source of heterogeneity in terms of the mean age, duration of the intervention, preparation time before the RAT, and previous TBL experienced by students.

**Conclusions:**

Students achieved significantly higher scores for the GRAT than for the IRAT, especially the group which only included nursing students, which implies excellent collaboration in the group of nursing students.

## Background

Problem-based learning (PBL) and team-based learning (TBL) are both commonly used instructional strategies in the medical field. They are implemented to enhance students’ clinical reasoning and knowledge acquisition by having them engage in small-group discussions [1, 2]. While both models embody the element of group discussions, problem-based learning focuses on the pedagogy of learning through problem-solving process and team-based learning features the collaboration aspect of the learning process. A major difference between PBL and TBL is the teacher-student ratio. In TBL model, one instructor can lead twenty or even more study teams simultaneously, whereas in PBL each small group requires one instructor’s facilitation. Therefore, TBL provides an attractive feature desired by the medical field [3, 4].

Furthermore, TBL reveals its strength in terms of learner involvement. Kelly, Haidet [5] conducted a study which observed engagement behaviors and found a higher learner-instructor engagement within TBL than PBL. A prerequisite of TBL is that participants must come prepared to every session, and each student is held accountable for their contribution to team performance [3]. With each of the steps carefully implemented, TBL shows a higher learner involvement without close supervision from the instructor. However, this notion does not hold true in PBL.

**Fig. 1:**
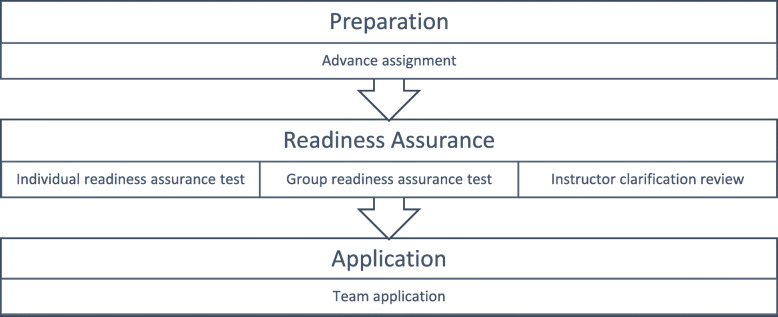
Flow chart of TBL process

To maintain students’ awareness of their accountability, a series of design decisions is needed to create an optimal environment for TBL. Hence, instructors’ design decisions must cover the entire instructional activity sequence, which embraces three main stages [3, 6, 7]. See Fig. 1.

1. Preparation: Adequate reading materials for the TBL session are assigned prior to the class to help students become acquainted with the learning objectives and key concepts [7].
2. Readiness assurance: The readiness assurance test (RAT) is a measure to determine whether each participant is prepared for the next application step of TBL [2]. This is an essential feature to differentiate TBL from PBL activity sequence. There are three sub-steps in this stage. First is the IRAT that requires each student to complete a set of multiple-choice questions (MCQs) to demonstrate individual understanding of the session content. After that, students are assigned to different learning groups to collaboratively answer the same set of MCQs together through the protocol of “consensus-building discussion” [3]. This is considered as group RAT (GRAT). After the team test, students have an opportunity to restore credit on the RAT, in terms of appeals. Students may request that the instructor an alternative answer to the one designated as the best. In the last step of this stage, the instructor gathers the groups together to provide feedback and address the misconceptions manifested in the previous sub-step.
3. Application of course concepts: In this last stage of team-based learning, students are given with a clinical case scenario and asked to make interpretations and analyses of the given information. Their task is to perform reasoning to make a diagnosis and propose possible treatment plans [7].

The principle of team-based learning is consistent with Vygotsky’s theory of social constructivism [7]. He believed that knowledge is co-constructed through natural social interactions where individuals learn from one another. Furthermore, his theory of the Zone of Proximal Development (ZPD) inspires the focus of this study. Vygotsky used ZPD to describe the difference between one’s independent problem-solving performance and his or her achievement with help from others, such as instructors or more advanced peers [8]. In this study, a TBL session includes the preview of the learning materials at the preparation stage and the quizzes at the readiness assurance stage that serve as scaffolds to support students’ learning progress [7]. We discovered that students were already able to demonstration progress as identified in the ZPD during GRAT, even before instructors’ guidance. Students’ improvement from the IRAT to the GRAT can be hence employed as their initial TBL performance. Gopalan, Fox [9] indicated that providing an IRAT before a GRAT could enhance students’ engagement, improve team performances, and reduce time needed to complete team assignments. According to a systematic review by Remschiel et al. [2], 52 of the 85 studies on TBL showed outcomes that the GRAT scores were higher than IRAT scores. However, variations may occur due to specific dynamics that affect member engagement within teams. For instance, team members’ social-emotional intelligence might have a direct impact on the quality of interactions [10]. Burgess, Ayton, and Mellis [11] showed that students’ GRAT scores were lower than their IRAT scores. Das et al. [12] investigated if gender was a variable that can affect TBL outcomes. They concluded that female students exhibited better achievement in tests that assessing problem solving skills than male students after TBL sessions. Their study also indicated that low achievers benefited from conventional lectures than from TBL.

Due to contradictory findings of the above-mentioned studies on learning outcomes of TBL, a meta-analysis of TBL performances is needed to clarify which outcomes of TBL can be expected, and which cannot. The purpose of this meta- analysis study was to examine the performance of students of healthcare professions on GRAT and IRAT during TBL. The hypothesis of this meta-analysis was that students would obtain a higher score on the GRAT than IRAT.

## Methods

### Study design

This meta-analysis followed guidelines of the Preferred Reporting Items for Systematic Reviews and Meta-Analyses: The PRISMA Statement [13].

### Literature search

Databases, including PubMed and Cochrane database were searched from inception to December 2018. After referring to Reimschisel’s systematic review [2] and Chen’s meta-analysis study [14], the following key words were applied: readiness assurance test AND team-based learning. All studies were included in the reference management software Endnote™ and studies that met the selection criteria were used for quality assessment and data extraction.

### Inclusion criteria

We used studies that met the following selection criteria: 1) the TBL instructional strategy was applied for healthcare professionals’ education with a clear source of participants; and 2) the study showed explicitly quantitative results for the GRAT and IRAT, and data of the mean and standard deviation (SD) were available. This meta-analysis used available data from 11 studies, comprising 1575 participants.

Each included study used the same scoring system for IRAT and GRAT. Articles with qualitative methods, review articles, and articles written in languages other than English were excluded.

### Data extraction and quality assessment

Two authors first independently vetted the search results and then concurred on the final relevant selection. As they compared their results, if a disagreement on a given study, it was again reviewed by a third investigator. After a discussion among the three investigators, they formed a consensus. These investigators extracted the following information from selected studies. See Table 1**.**

**Table 1:** Characteristics of the included studies

First author and year publication	Disciplines	Study design	TBL participants and F: M ratio	Source of participants	Student performance	Scoring system for the RAT
Cheng CY (2014) [15]	Single discipline	Cohort study (Pre-post-test)	387 F: M N/A	adult health nursing, maternal-child nursing, community health nursing, medical-surgical nursing students	IRAT 64.32 ± 12.71 GRAT 88.65 ± 5.52	Percentage
Farland MZ (2018)	Single discipline	Cohort study (retrospective)	442 F: M N/A	pharmacy resident students	IRAT 16.18 ± 2.45 GRAT 18.80 ± 1.68	Ranged 0 ~ 20 points
Goolsarran N (2018) [16]	Multidisciplinary	Cohort study	76 F: M N/A	internal medicine intern and senior nursing students	IRAT 5.6 ± 1.7 GRAT 7.7 ± 1.8	Ranged 0 ~ 10
Hemmati Maslakpak M (2015)	Single discipline	Quasi-experimental study (TBL in intervention group; Lecture in control group)	32 F: M 16: 6	third year nursing students	IRAT 25.05 ± 3.36 GRAT 31.68 ± 1.33	N/A
Huang Z (2016)	Single discipline	Cohort study	99 F: M N/A	clinical medicine program students	IRAT 63.78 ± 9.30 GRAT 75.65 ± 7.40	Percentage
Lochner L (2018)	Multidisciplinary	Cohort study	39 F: M N/A	nursing, dietetics and nutrition, occupational therapy, radiology techniques, laboratory techniques students	IRAT 10.59 ± 0.65 GRAT 14 ± 0.5773	Ranged 0 ~ 14 points
Luetmer MT (2018) [17]	Multidisciplinary	Cohort study	81 F: M N/A	first year medical and physical therapy students	IRAT 69.9 ± 8.6 GRAT 95.2 ± 10.2	Percentage
Nishigawa K (2017)	Single discipline	Cohort study	256 F: M N/A	third- and fourth-year dental students	IRAT 63.1 ± 13.7 GRAT 77.8 ± 9.9	Percentage
Park HR (2015) [18]	Single discipline	Cohort study	74 F: M 68: 6	second-year nursing students	IRAT 80.47 ± 10.76 GRAT 96.44 ± 2.23	Percentage
Park SE (2018)	Single discipline	Cohort study	34 F: M N/A	second year dental students	IRAT 49.87 ± 16.045 GRAT 87.95 ± 8.345	Percentage
Zeng R (2017) [19]	Single discipline	Randomized Controlled Trails (TBL in intervention group; Lecture in control group)	55 F: M 27: 28	third year medical undergraduates	IRAT 16.56 ± 3.89 GRAT 25 ± 1.05	N/A

The quality of the studies included was assessed by the Newcastle-Ottawa (NO) Quality Assessment Scale of Cohort Studies [20]. The full score of the NO Scale is 9 points, and a study awarded 6 points was considered a high-quality study.

For one randomized control trial we used the Cochrane Risk of Bias Tool for Randomized Controlled Trials (RCTs) and examined the following items: randomization, allocation concealment, selective reporting, blinding, outcome assessment, and incomplete outcome data.

### Statistical analysis

The difference between GRAT and IRAT scores was assessed, and subgroup analyses to investigate sources of heterogeneity according to study characteristics [21] were performed with Review Manager (RevMan) vers. 5.3 [22].

The publication bias evaluation, including Egger’s test, Begg’s test, and a meta-regression, was performed with Comprehensive Meta-Analysis version 3 [23].

## Results

### Study characteristics and quality assessment

The study selection process of all eligible studies is presented in Fig. 1. In total, 21 studies in English were retrieved from the PubMed and Cochrane databases, and two duplicate studies were excluded. After screening the title and abstract, 19 studies were retained for further assessment; we excluded two studies which were review articles and one study that was written in Korean. Following evaluation using the inclusion criteria, 5 articles were removed since there was no available quantitative information related to the IRAT and GRAT. One study conducted by Burgess, Ayton [11] was also excluded, because the GRAT and IRAT scores could not be compared to each other, as this study reported students’ GRAT and IRAT scores using two different scoring systems. In total, 11 studies including 1575 participants were identified.

Regarding inter-professional education, participants in eight of these 11 studies were from the same discipline such as school of medicine, nursing or pharmacy. The total number here is not equal to the 11 studies based on criteria selection in this meta-analysis, since students that performed TBL were also recruited. Six of the eleven studies used a percentage system for scoring RAT achievements. The other five studies used different score systems for RAT. The original RAT scores were therefore converted to the value of standardized mean difference for further RAT scores comparison. We found that three studies showed different full scores (in ranges of 0 ~ 10, 0 ~ 14, and 0 ~ 20). However, two studies did not reveal their RAT scoring systems. The ratio of gender in these 11 studies were not fully disclosed, with only three of 11 studies revealing their female to male ratios.

Table 2 shows the methodological quality of ten included studies according to the NO Scale for cohort and case control studies. Quality scores ranged 4 ~ 11. Six of the ten studies were determined to be of fair quality, and four studies were considered to be of good quality.

**Table 2:** Methodological quality of the included studies

Study	Selection	Comparability	Outcomes	Total score	Quality of studies
Cheng et al. (2014) [15]	★★	★	★★★	6	Good
Farland et al. (2018)	★★	★	★★★	6	Fair
Goolsarran et al. (2018) [16]	★★★		★	4	Fair
Hemmati Maslakpak et al. (2015)	★★★	★	★★★	7	Good
Huang et al. (2016)	★★★	★	★★	6	Good
Lochner et al. (2018)	★★	★	★★	5	Fair
Luetmer et al. (2018) [17]	★★★		★★★	6	Fair
Nishigawa et al. (2017)	★★	★	★★★	6	Fair
Park et al. (2015) [18]	★★	★	★★	5	Fair
Park et al. (2018)	★★★	★	★★★	7	Good

According to Newcastle – Ottawa quality assessment scale for cohort and case control studies, each study can be awarded a maximum of one star for each item within the “Selection” and “Outcome” categories, and two stars in maximum for “Comparability”

We used the Cochrane Risk of Bias Tool for Randomized Controlled Trials to assess the quality of one RCT. A study conducted by Zeng et al. [19] was assessed as having fair quality, with a low risk of randomization, selective reporting, blinding, outcome assessment, and incomplete outcome data and an unclear risk of allocation concealment.

### Data synthesis

**Fig. 2:**
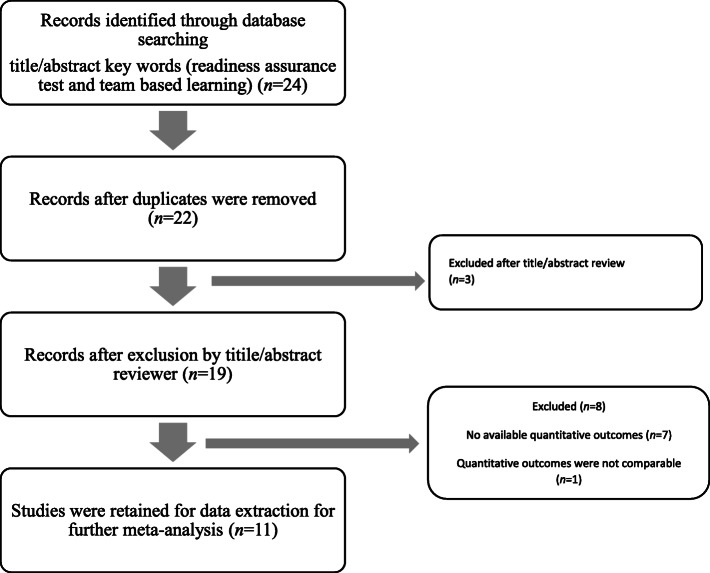
Flow chart of inclusion of studies for the meta-analysis

Since the scores of the RAT were reported in all 11 studies, differences between the GRAT and IRAT scores were pooled in this meta-analysis. In Fig. 2, the GRAT scores were statistically greater than IRAT scores when TBL was applied in a random-effects model (standardized mean difference (SMD) = 2.05, 95% confidence interval (CI) = 1.64 ~ 2.46, *p*_heterogeneity_ < 0.001 *I*^2^ = 95%).

Begg’s test provided an insignificant result (*p* = 0.11), which means that the funnel plot was not asymmetrical. Therefore, publication bias was less likely to have occurred. Egger’s test gave a one-tailed *p* value of 0.045 and a two-tailed *p* value of 0.089.

### Test of heterogeneity

**Fig. 3:**
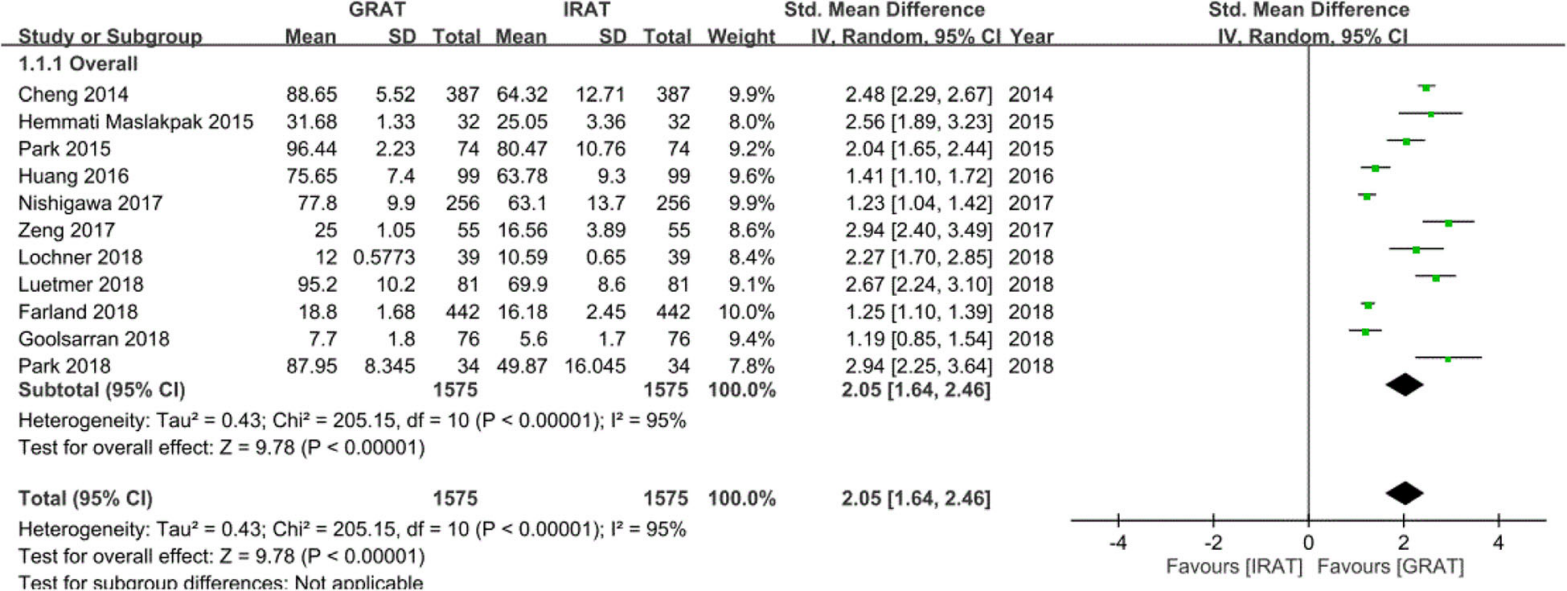
Forest plot of differences in the group readiness assurance test (GRAT) and individual readiness assurance test (IRAT) scores

When conducting the subgroup analysis, the group that comprised only nursing students showed a higher SMD of 2.368, 95% CI = 2.05 ~ 2.6, *p*_heterogeneity_ =0.14, and *I*^2^ = 48% (Fig. 3).

**Fig. 4:**
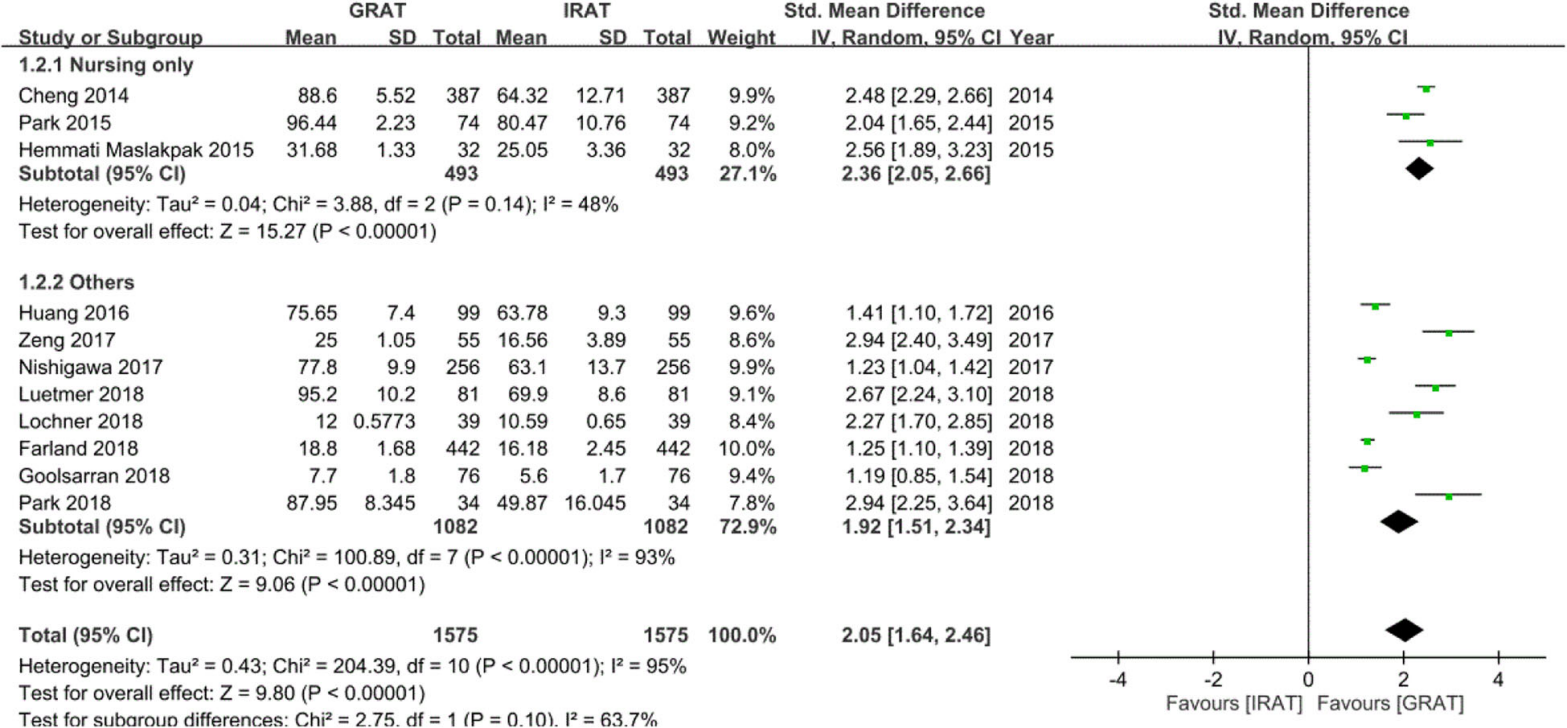
Forest plot of differences in the group readiness assurance test (GRAT) and individual readiness assurance test (IRAT) scores in the subgroup analysis between only nursing students and others

The others group presented a lower difference in GRAT and IRAT scores with SMD = 1.92, 95% CI = 1.51 ~ 2.3, *p*_heterogeneity_ < 0.001, and *I*^2^ = 93% (Fig. 3).

There were no statistically significant subgroup differences (*p* = 0.1 in Fig. 4). However, smaller numbers of studies and participants contributed data to the nursing-only subgroup than to the others subgroup, such that the analysis may have been less likely to detect a subgroup effect.

The percentage of heterogeneity between results from studies within the nursing-only subgroup decreased from 93 to 48% (Fig. 4). The subgroup analysis explained the heterogeneity in the overall analysis, because the heterogeneity within the subgroups was lower than the heterogeneity among the eleven studies. The meta-regression indicated that the quality score and sample size were less likely to have caused the heterogeneity (Table 3).

**Table 3:** Meta-regression analysis of the sources of heterogeneity

Factor	Number of studies	Coefficient	Standard error	95% confidence interval	*P* value
Quality score	11	0.354	0.27	0.15 ~ 0.86	0.17
Sample size	11	−0.002	0.002	0.005 ~ 0.001	0.21

## Discussion

This meta-analysis extracted available data of 11 empirical studies from 24 studies on TBL, comprising 1575 participants. Our meta-analysis found that students achieved significantly higher scores on the GRAT than the IRAT among different majors in medical education. Based on the guidance of TBL [3, 6, 7], students are required to finish the IRAT before taking the GRAT. The improvement from the IRAT to the GRAT could be regarded as the learning and progress occur in the Zone of Proximal Development, as the result of students collaborating with one another [9]. Gopalan et al. (2013) reported that it took less time for students to complete their assignments in sections including both the IRAT and GRAT, compared to the group of students only taking the GRAT. This finding confirms the importance of IRAT as the measure to establish individual accountability and to ensure the effectiveness of TBL.

According to our subgroup analysis, the group consisting of only nursing students achieved much higher improvement with the GRAT than with the IRAT (SMD = 2.368, 95% CI = 2.065 ~ 2.670, *p*_heterogeneity_ 0.14, *I*^2^ = 48.77) compared to the non-nursing group although *p* = 0.1 (Fig. 3) may have been due to the limited number of studies in the two subgroups. This is because TBL is being newly applied in nursing education, and few studies have been published on implementing TBL in nursing education [15]. We could not find other reviews or studies that conducted subgroup analyses with only nursing students and others, and thus this study can be considered the first one to conduct this type of subgroup analysis. Two assumptions were raised to explain this finding:

First, the vast majority of nurses are females. It was reported that males and females interact differently in learning situations [24], and research found that females perform better with collaborative learning [25]. Atlasi, Moravveji [26] indicated that female students were more motivated to learn in small groups than male students. Wehrwein, Lujan [27] reported that only 4.2% of male students preferred learning by reading printed texts and writing compared to other modes of instruction. Thus, the preferred learning styles between males and females may have affected outcomes of TBL. Three studies of this meta-analysis enrolled only nursing students. Only two of these three studies revealed the ratio of gender in their population. Part et al. (2015) reported that the female-male ratio of their study was 13.3:1, while Hemmati Maslakpak (2015) had a 1:1 female-male ratio in his study. Female students were the majority in one study and were equal to male students in the other; thus this could have been a confounding variable affecting TBL outcomes. Since female participants were also enrolled in the other eight studies, gender as the reason to explain why nursing students perform better does not hold true.

The second assumption to explain this subgroup analysis finding is that nursing students collaborate better than students in other professions because the level of professionalism required might be greater in nursing. Burford et al. [10] discovered that medical students’ readiness for inter-professional learning and collaboration was high initially, but declined significantly over time. Meanwhile, nursing students had consistently higher scores of readiness for inter-professional learning and group identification than the medical students. Studies also suggest that nursing students often show more-positive attitudes toward teamwork than medical or dental students [28, 29]. However, even with these probable assumptions, we could not find evidence to confirm this notion from the included 11 studies.

In this meta-analysis, we found no correlations among variables, including the quality score, sample size, and SMD between the IRAT and GRAT of these 11 studies (Table 3). Thus, the SMD between the GRAT and IRAT could not be predicted by the quality score or by the sample size of these studies. The included 11 TBL is applicable in classes with either small or large enrollments. As mentioned in the introduction, compared to PBL, TBL allows a single instructor to simultaneously handle a large number of students through teamwork, while PBL requires multiple faculty members and rooms for each panel session [3, 4]. The findings of the current study are consistent with those of Kibble et al. (2016), who explored critical elements for TBL curriculum design and implementation with effective classroom management in mind. However, that study also suggested that classroom resources, and classroom management strategies and logistics are essential to adequately prepare when applying TBL in larger classes [30].

Limitations of this meta-analysis stem from individual studies. These 11 studies insufficiently revealed other related factors, including the mean age, duration of conducting TBL, time preparation for the RAT, team size, and prior formal experience of TBL participants. Thus, we could not use the meta-regression to explore the source of heterogeneity. Second, various scoring systems were conducted in these collected studies. Some studies used a percentage-based system [15, 18], Luetmer, Cloud [17], while others used other scoring systems [16]. The SMD was hence used in this meta-analysis to detect differences between the GRAT and IRAT. Our study suggests the need for official guidelines for scoring systems for TBL. Future studies can compare performances of the RAT between nursing students and other interdisciplinary groups.

## Conclusions

Although PBL and TBL both can engage students in the discourse of solving clinical problems, tutors of TBL are not able to guide each intra-group discussion regarding high teacher-pupil ratio. Cooperative learning among peers is much more demanded in TBL. This study investigated hence whether TBL participants performed better after group discussions than their own individual learning achievements in terms of RAT scores. Results indicated that students’ GRAT scores, i.e., cooperative learning achievements, were significantly higher than their individual test (IRAT) scores. Based on a subgroup analysis, nursing students exhibited greater differences than students of other health professions in RAT scores after group discussions. Whether this is relating to the nature of nursing profession, further research is much needed. The implementation of TBL was shown in many studies; however, how the IRAT and GRAT are scored need to be clearly stated. Related factors, including the mean age, duration of the intervention, and preparation times before RAT and formal TBL experienced by students, should also be considered. Future studies can compare performances of the RAT between nursing and other interdisciplinary TBL settings.

## Data Availability

All raw data used in this systematic review and meta-analysis were extracted from available published articles.

## Abbreviations

PBL: Problem-based Learning
TBL: Team-based Learning
RAT: Readiness Assurance Test
IRAT: Individual Readiness Assurance Test
GRAT: Group Readiness Assurance Test
ZPD: Zone of Proximal Development
RCT: Randomized Controlled Trials
PRISMA: Preferred Reporting Items for Systematic Reviews and Meta-Analyses
